# Impact of Early Versus Late Antiretroviral Treatment Initiation on Naive T Lymphocytes in HIV-1-Infected Children and Adolescents – The-ANRS-EP59-CLEAC Study

**DOI:** 10.3389/fimmu.2021.662894

**Published:** 2021-04-22

**Authors:** Pierre Frange, Thomas Montange, Jérôme Le Chenadec, Damien Batalie, Ingrid Fert, Catherine Dollfus, Albert Faye, Stéphane Blanche, Anne Chacé, Corine Fourcade, Isabelle Hau, Martine Levine, Nizar Mahlaoui, Valérie Marcou, Marie-Dominique Tabone, Florence Veber, Alexandre Hoctin, Thierry Wack, Véronique Avettand-Fenoël, Josiane Warszawski, Florence Buseyne

**Affiliations:** ^1^ Immunologie, hématologie et rhumatologie pédiatrique, hôpital Necker–Enfants malades, AP–HP- Centre – Université de Paris, Paris, France; ^2^ Laboratoire de microbiologie clinique, hôpital Necker–Enfants malades, AP–HP-Centre – Université de Paris, Paris, France; ^3^ EHU 7328 PACT, Institut Imagine, Université de Paris, Paris, France; ^4^ Unité Epidémiologie et Physiopathologie des Virus Oncogènes, Institut Pasteur, Paris, France; ^5^ Département de Virologie, UMR CNRS 3569 Institut Pasteur, Paris, France; ^6^ Départment d’épidémiologie, Centre de Recherche en Épidémiologie et Santé des Populations, INSERM U1018, Le Kremlin-Bicêtre, Villejuif, France; ^7^ Hémato-oncologie pédiatrique, Hôpital Trousseau, AP-HP, Paris, France; ^8^ Pédiatrie Générale, Hôpital Robert Debré, AP-HP, Paris, France; ^9^ Pédiatrie et néonatologie, Centre hospitalier intercommunal de Villeuneuve-Saint-Georges, Villeuneuve-Saint-Georges, France; ^10^ Pédiatrie Générale, Hôpital Bicêtre, AP-HP, Paris, France; ^11^ Pédiatrie Générale, Centre hospitalier intercommunal de Créteil, Créteil, France; ^12^ Immuno-hématologie pédiatrique, Hôpital Robert Debré, AP-HP, Paris, France; ^13^ Médecine et réanimation néonatale, Hôpital Cochin, AP-HP-Centre – Université de Paris, Paris, France; ^14^ CNRS 8104/INSERM U1016, Institut Cochin, Université Paris Descartes, Paris, France; ^15^ INED, Université Paris Sud, Le Kremlin-Bicêtre, Orsay, France

**Keywords:** HIV-1, children, adolescents, early ART, T lymphocyte, naive T lymphocyte

## Abstract

**Background:**

The early initiation of antiretroviral therapy (ART) in HIV-1-infected infants reduces mortality and prevents early CD4 T-cell loss. However, the impact of early ART on the immune system has not been thoroughly investigated in children over five years of age or adolescents. Here, we describe the levels of naive CD4 and CD8 T lymphocytes (CD4/CD8T_N_), reflecting the quality of immune reconstitution, as a function of the timing of ART initiation (early (<6 months) versus late (≥24 months of age)).

**Methods:**

The ANRS-EP59-CLEAC study enrolled 27 children (5-12 years of age) and nine adolescents (13-17 years of age) in the early-treatment group, and 19 children (L-Ch) and 21 adolescents (L-Ado) in the late-treatment group. T lymphocytes were analyzed by flow cytometry and plasma markers were analyzed by ELISA. Linear regression analysis was performed with univariate and multivariate models.

**Results:**

At the time of evaluation, all patients were on ART and had a good immunovirological status: 83% had HIV RNA loads below 50 copies/mL and the median CD4 T-cell count was 856 cells/µL (interquartile range: 685-1236 cells/µL). In children, early ART was associated with higher CD8T_N_ percentages (medians: 48.7% vs. 31.0%, *P* = 0.001), and a marginally higher CD4T_N_ (61.2% vs. 53.1%, *P* = 0.33). In adolescents, early ART was associated with low CD4T_N_ percentages and less differentiated memory CD8 T cells. CD4T_N_ and CD8T_N_ levels were inversely related to cellular activation and gut permeability.

**Conclusion:**

In children and adolescents, the benefits of early ART for CD8T_N_ were clear after long-term ART. The impact of early ART on CD4T_N_ appears to be modest, because pediatric patients treated late respond to HIV-driven CD4 T-lymphocyte loss by the *de novo* production of T_N_ cells in the thymus. Our data also suggest that current immune activation and/or gut permeability has a negative impact on T_N_ levels.

**Clinical Trial Registration:**

ClinicalTrials.gov, identifier NCT02674867.

## Introduction

HIV-1-infected children receiving antiretroviral treatment (ART) face a lifetime of chronic disease due to this virus (1–3). Early ART initiation has an immediate beneficial impact, by blocking clinical progression (4), decreasing the cell-associated viral reservoir in infants (5) and favoring faster CD4 T-lymphocyte recovery (1, 6, 7). However, the benefit of early ART for CD4 T-cell levels may decrease after longer periods on ART. Early ART initiation may preserve naive CD8 T lymphocytes (CD8T_N_) (8) and CD4/CD8 ratio (9). Early ART reduces the frequency of HIV-specific CD4 and CD8 T cells in children, but preserves the capacity of these cells to produce several cytokines (10–12). Overall, findings concerning the impact of early ART on CD4 and CD8 T cells in pediatric patients remain limited.

Several gaps in our knowledge of the long-term impact of early versus late ART remain. Few studies have investigated the immunological benefits of early ART initiation in children over the age of five years and those without sustained viral suppression. The benefit of early ART for CD4 T-cell levels may persist or decrease over longer periods of long-term effective ART. Finally, unplanned treatment interruptions frequently occur during early childhood and adolescence, and it remains unclear whether the deleterious impact of viral rebound on the benefits of early ART is transient or irreversible (13–15).

The ANRS-EP59-CLEAC study investigated the immunological and virological characteristics of HIV-1-infected children over five years of age and adolescents, as a function of age at ART initiation (< 6 months *vs.* ≥ 24 months of age). We included participants with an initial period of viral suppression but without any criteria on the persistence of viral control (16). Here, we focused on the proportions of CD4T_N_ and CD8T_N_, the major indicators of qualitative immune reconstitution (17), and their relationships with current and past HIV disease parameters and current immune activation (3).

## Patients and Methods

### Patients

The ANRS-EP59-CLEAC study was conducted in accordance with the Helsinki Declaration and the protocol was approved by the “Comité de protection des personnes île-de-France V”. Agreement to participate was obtained from the participants, if they were old enough to give an opinion, and written informed consent was obtained from at least one parent. The main inclusion criteria were (1) HIV-1 infection after vertical transmission, (2) participant aged 5 to 17 years at the time of the study, (3) ART initiation for therapeutic purposes before six months of age or after two years of age, (4) with initial virological success (HIV-1 RNA < 400 copies/mL achieved within 24 months of treatment initiation), regardless of the subsequent course of viremia. Between 2016 and 2019, two 15 mL blood samples were collected for biological evaluations, at two consecutive routine clinical follow-up visits.

### Biological Evaluations

Total blood cell-associated HIV-1 DNA was quantified by ultrasensitive real-time PCR (18). The CD4 and CD8 T-lymphocyte subsets were quantified in fresh blood by flow cytometry with combinations of antibodies targeting the CD3, CD4, CD8α, CD45RA, CCR7, CD27, CD28, CD31, CD95, HLA-DR, and CD38 molecules(**Supplementary Material**). Data were collected on a Gallios cytometer (Beckman Coulter) and analyzed with Kaluza software (Beckman Coulter). The gating strategy involved the sequential definition of lymphocytes, CD4 and CD8 T lymphocytes, and their subsets according to CD45RA and CCR7 expression. The subsets were further characterized by using CD27 and CD28 expression to define naive (T_N_), central memory (T_CM_), transitional memory (T_TM_), effector memory (T_EM_) and effector (T_E_) cells; CD31 and CD95 expression was used to define recent thymic emigrants (T_RTE_) and stem cell memory cells (T_SCM_); and HLA-DR and CD38 expression was used to define activated T cells (**Supplementary Material**).

C-reactive protein (CRP), interleukin-6 (IL-6), CXCL10, soluble CD14 (sCD14), soluble CD163 (sCD163), and intestinal fatty acid-binding protein (iFABP) were quantified by ELISA (**Supplementary Table 1**). The Liaison XL CMV IgG (Diasorin) chemiluminescence immunoassay and the CMV R-Gene (Biomérieux) PCR assay were used to quantify cytomegalovirus (CMV) antibodies and DNA in plasma samples. The frequency of interferon (IFN)-γ-producing cells was quantified in an Elispot assay with anti-IFN-γ capture/detection antibodies (Diaclone 869.060.010), streptavidin alkaline phosphatase conjugate (GE Healthcare RPN4402) and BCIP/NBT color development substrate (S3771, Promega), according to the kit manufacturers’ instructions. Fresh peripheral blood mononuclear cells (PBMCs) were used to seed High Protein-Binding Immobilon-P Membrane P96 plates (MSIPS4510, Merk Millipore) at a density of 2 x 10^5^ and 5 x 10^4^ cells/well in RMPI supplemented with 10% fetal calf serum, with the addition of pp65 CMV peptides (#11549, NIH AIDS Reagent Program, 1 µg/mL of each peptide) or peptide diluent (DMSO). The cells were then cultured for 24 h. Spot-forming cells (SFCs) were counted with an ImmunoSpot S6 UV Image Analyzer (CTL, Bonn, Germany).

### HIV History Variables

The patients were either included at birth in The Agence Nationale de Recherche sur le SIDA et les Hépatites Virales (ANRS) EPF/ANRS CO10 national prospective multicenter cohort or were managed at the centers of the ANRS/CO10 cohort and diagnosed before the age of 13 years (1). Clinical and biological data were therefore collected for HIV-infected children *via* the completion of standardized questionnaires at six-month intervals, or were collected retrospectively after inclusion in the CLEAC study. All ART regimens initiated consisted of highly active antiretroviral treatment (HAART; i.e., any combination of at least three different antiretroviral drugs or any combination including one protease inhibitor, or one non-nucleoside reverse transcriptase inhibitor or one integrase inhibitor). During the first few weeks of life, 30 children received prophylactic ART consisting of zidovudine (*n*=21), zidovudine+lamivudine (*n*=5), zidovudine+lamivudine+lopinavir (*n*=2) or zidovudine+lamivudine+nevirapine (*n*=2). For the last four of these children, the date of first HAART (ART1) initiation was the date of prophylactic ART initiation, as treatment was continued without interruption after diagnosis. At the time of the study, all but one of the children were on HAART, the remaining child being on two nucleoside reverse transcriptase inhibitors and having an undetectable viral load.

CD4 and CD8 T cells and plasma HIV RNA levels were quantified at each clinical site. The threshold values used in HIV RNA assays depended on study site, and a value of 50 copies/mL was selected as the common cutoff for current HIV RNA detection. A cutoff of 400 copies/mL was used for the assessment of virological history, because of changes in HIV RNA quantification assays and cutoffs over time. Cumulative viremia was defined as the area under the HIV RNA curve over time (19). Past immunological parameters were defined using CD4 T-cell percentages, because the age-related variations of this parameter are smaller than those for CD4 T-cell counts. The duration of viral suppression and cumulative viremia were assessed from ART1 initiation. These parameters were normalized by dividing their values by the time since ART1 initiation.

### Statistical Analysis

Analyses were stratified according to age at evaluation (children: 5-12 years of age; adolescents: 13-17 years of age). Wilcoxon-Mann-Whitney, Kruskal-Wallis and the Fisher’s exact tests were used to compare the characteristics of children and adolescents between the early and late treatment initiation groups. The corrplot package of R was used to display Spearman’s rank correlation coefficients. Univariate and multivariate analyses were performed by linear regression, with CD4/CD8T_N_ percentages as the continuous dependent variables. Multivariate models included early ART as the main independent variable, together with noncollinear variables with *P* values <.2 in univariate analysis. Most models were built with a single biological variable, to prevent over-adjustment. Observations with high leverage values or studentized residuals were excluded to test the robustness of the models. Analyses were conducted with SAS statistical software. *P* values <.05 were considered statistically significant.

## Results

### Patient Characteristics

We prospectively enrolled 27 children (E-Ch) and nine adolescents (E-Ado) in the early ART (E) group, and 19 children (L-Ch) and 21 adolescents (L-Ado) in the late ART (L) group (**Table 1**). Just over half (54%) of the patients were girls. Most patients (74%) were born to mothers originating from Sub-Saharan Africa. However, 60% of patients were born in mainland France, and ART was initiated early more frequently in these children than in those born abroad. At the time of the study, children were more frequently aviremic (89% vs. 73%, *P* = .07), and had higher CD4 T-cell counts than adolescents (median ([IQR]: 954 [745;1320] vs. 766 [622;1092] cells/µL, *P* = .04). Immunovirological status was good in most patients at the time of the study.

**Table 1:** Patient’s characteristics.

Characteristics^a^	Children (5-12 years)		Adolescents (13-17 years)		All	*P* value^a^
	Early treatment	Late treatment	Early treatment	Late treatment		
	*n* = 27	*n* = 19	*n* = 9	*n* = 21	*n* = 76	
Age, years	9 [6;11]	8 [7;10]	15 [14;16]	14 [13;15]	11 [8;14]	.0001
Sex Male Female	33.3 (9) 66.7 (18)	42.1 (8) 57.9 (11)	44.4 (4) 55.6 (5)	66.7 (14) 33.3 (7)	46.0 (35) 54.0 (41)	.14
Sub-Saharan African origin No Yes	25.9 (7) 74.1 (20)	26.3 (5) 73.7 (14)	22.2 (2) 77.8 (7)	23.8 (5) 76.2 (16)	25.0 (19) 75.0 (57)	1.00
Born in mainland France No Yes	7.4 (2) 92.6 (25)	73.7 (14) 26.3 (5)	0.0 (0) 100.0 (9)	71.4 (15) 28.6 (6)	40.8 (31) 59.2 (45)	<.0001
Age at ART1 initiation (months)	2.1 [0.5;3.4]	54.3 [49.9;81.4]	2.3 [1.7;2.7]	93.0 [55.4;137.3]	25.2 [2.3;79.0]	.0001
Time since ART1 initiation (months)	119 [75;137]	52 [23;70]	188 [178;198]	85 [40;124]	93 [57;137]	.0001
Current HIV RNA <50 copies/ml ≥50 copies/ml	88.9 (24) 11.1 (3)	89.5 (17) 10.5 (2)	66.7 (6) 33.3 (3)	76.2 (16) 23.8 (5)	82.9 (63) 17.1 (13)	.30
CD4 count, cells/µL	951 [725;1320]	1009 [745;1527]	840 [713;1078]	740 [622;1092]	856 [685;1236]	.20
CD4 percentage	39 [37;44]	37 [33;41]	39 [34;44]	36 [29;41]	38 [32.5;42]	.18
CD8 count, cells/µL	574 [478;876]	939 [724;1035]	675 [539;767]	672 [510;1016]	714 [519.5;985]	.02
CD8 percentage	26 [22;31]	31 [27;36]	30 [25;43]	31 [26;42]	29 [25;36]	.07
CD4/CD8 ratio	1.57 [1.10;1.90]	1.26 [0.89;1.48]	1.34 [0.92;1.80]	1.06 [0.76;1.67]	1.34 [0.92;1.76]	.10
Lymphocyte count, 10^3^ cells/µL	2.4 [2.0;2.9]	2.8 [2.5;3.6]	1.8 [1.4;2.3]	2.0 [1.7;2.6]	2.4 [1.8;2.8]	.003
CD4T_N_ %	61.2 [49.9;68.9]	53.1 [50.0;58.5]	36.0 [33.2;48.9]	51.5 [43.1;59.1]	54.0 [46.4;62.5]	.01
CD8T_N_ %	48.7 [35.9;55.8]	31.0 [21.9;36.7]	29.0 [15.9;32.0]	29.0 [20.2;37.6]	35.1 [24.1;45.9]	.0002

a For categorical variables, frequencies, counts and Fisher’s exact test P values are indicated; for quantitative variables, median, interquartile range and Kruskal-Wallis’ test P values are indicated.

ART1, first highly active antiretroviral therapy; CD4T_N_ %, naive CD4 T lymphocyte percentage; CD8T_N_ %, naive CD8 T lymphocyte percentage.

### Early ART Was Not Significantly Associated With Higher CD4T_N_ Percentages in Children, and Was Associated With Lower CD4T_N_ Percentages In Adolescents

Blood CD4T_N_ percentages were not significantly higher in E-Ch than in L-Ch (medians: 61.2% vs. 53.1%, *P* = .33). E-Ado had significantly lower CD4T_N_ than L-Ado (36.0% vs. 51.5%, *P* = 0.02). The interaction between age and treatment group was significant (*P* = .008) and we performed analyses separately for children and adolescents.

**Figure 1:**
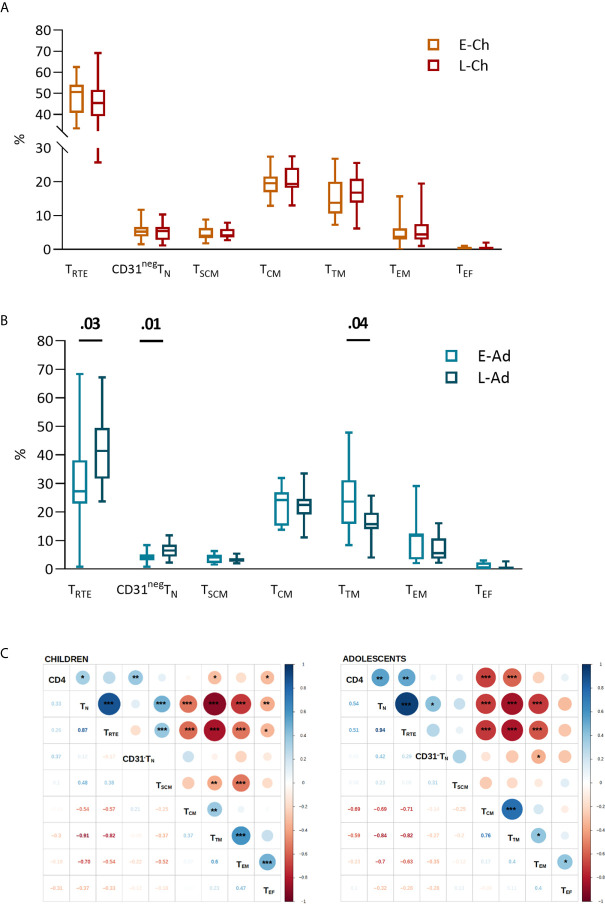
CD4 T-cell subsets in patients treated early and late. T-cell phenotypes were assessed by flow cytometry on fresh whole blood (**Supplementary Material**). The percentage of each subset among total CD4 T lymphocytes is presented for children **(A)** and adolescents **(B)**, on box and whiskers plots showing the minimum and maximum values. The early and late treatment groups were compared in Mann-Whitney tests; when significant, *P* values are shown on the graph. Subsets are presented from the least to the most differentiated, and were defined as naive T_N_ (CD45RA^+^CCR7^+^); recent thymic emigrants, T_RTE_ (CD45RA^+^CCR7^+^CD31^+^); CD31^neg^T_N_ (CD45RA^+^CCR7^+^CD31^-^); central memory, T_CM_ (CD45RA^-^CCR7^+^); transitional memory T_TM_ (CD45RA^-^CCR7^-^CD27^+^); effector memory T_EM_ (CD45RA^-^CCR7^-^CD27^-^) and effector T_EF_ (CD45RA^+^CCR7^-^CD27^-^CD28-) cells. **(C)** The correlograms present Spearman’s rank correlation coefficients as symbols (upper quadrants) and values. Significant associations are indicated by symbols (*p < .05; **p < .01; ***p < .001). CD4 T-cell counts were used for correlation analyses.

High interindividual variation in the distribution of CD4 T-cell subsets was observed in all groups (**Figures 1A, B**). No significant differences were observed between the E-Ch and L-Ch groups (**Figure 1A**). E-Ado had significantly lower levels of recent thymic emigrants (T_RTE_, 27.2% *vs.* 41.4%, *P* = .03) and CD31^neg^T_N_ (4.0% *vs.* 6.5%, *P* = .01), and higher levels of transitional memory (T_TM_) cells (26.6% *vs.* 15.7%, *P* = .04) than L-Ado. CD4 T-cell counts were positively correlated with CD4T_N_ and CD4T_RTE_, and negatively correlated with the levels of most memory subsets (**Figure 1C**).

**Figure 2:**
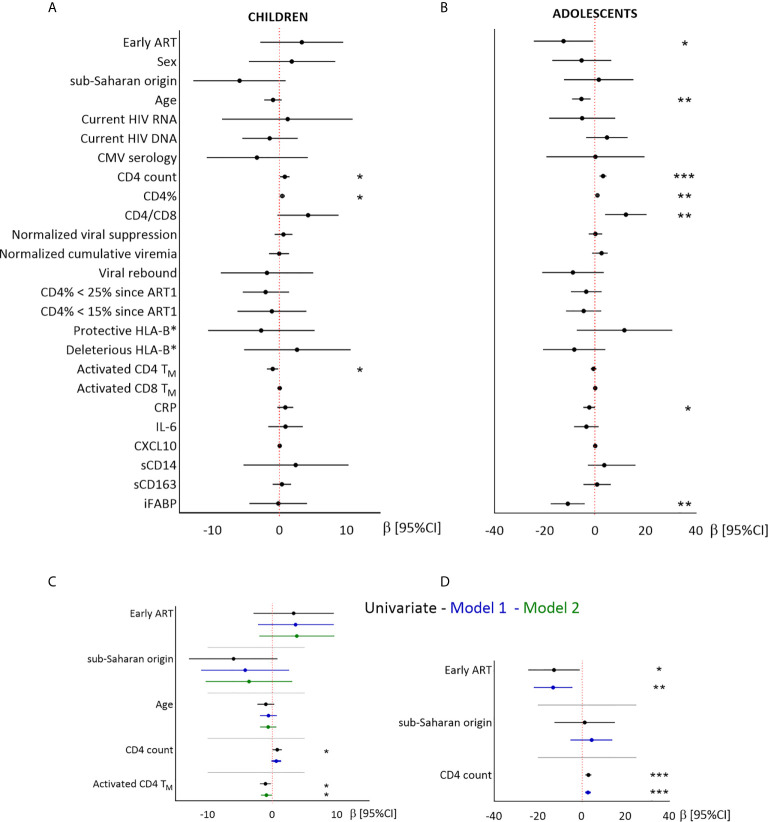
Linear regression analysis of the associations between CD4T_N_ and demographic, virological and immunological factors in children and adolescents. Results from univariate **(A, B)** and multivariate **(C, D)** linear regressions are presented as estimates (β) and 95% confidence intervals. Significant associations are indicated by symbols (*p < .05; **p < .01; ***p < .001). Multivariate analysis included the covariables indicated on the plot. A and C: children; B and D: adolescents. Estimates are given per year, 100 CD4 T cells, and 1% activated CD4T_M_.

We then searched for factors related to CD4T_N_ levels, including demographic factors, immunovirological status at the time of the study and during the time since ART1 initiation. Biomarkers of immune activation (activated CD4/CD8T_M_, sCD14, sCD163, and CXCL10), inflammation (CRP and IL-6) and gut permeability (iFABP) (**Figure 2** and **Supplementary Table 2**) were assessed in the samples used for T_N_ quantification.

In children, higher CD4T_N_ percentages were associated with higher CD4 T-cell counts and lower activated CD4T_M_ percentages (**Figure 2**). Sex and geographic origin were not significantly associated with CD4T_N_ percentages, but children of sub-Saharan origin tended to have lower CD4T_N_ percentages than other children (56.5% vs 62.5%, *P* = 0.08, **Figure 2** and **Supplementary Table 2**). After adjustment for treatment group, geographic origin and age, CD4T_N_ percentage was not associated with CD4 T-cell counts (model 1), but was inversely correlated with activated CD4T_M_ percentage (model 2).

In adolescents, higher CD4T_N_ percentages were associated with late treatment, younger age, higher CD4 T-cell counts, and lower plasma CRP and iFABP levels, but not with either sex or geographic origin (**Figure 2** and **Supplementary Table 2**). In multivariate analysis, higher CD4T_N_ percentages were associated with late treatment and higher CD4 T-cell counts (model 1). Multivariate models including other variables were not robust to outliers (data not shown).

In conclusion, early ART was not significantly associated with higher CD4T_N_ percentages in children, and was associated with lower CD4T_N_ percentages in adolescents. In both groups, CD4T_N_ percentages were associated with CD4 T-cell count and levels of immune activation and/or intestinal permeability.

### Early ART Is Associated With Higher Naive CD8T_N_ Percentages in Children but Not in Adolescents

**Figure 3:**
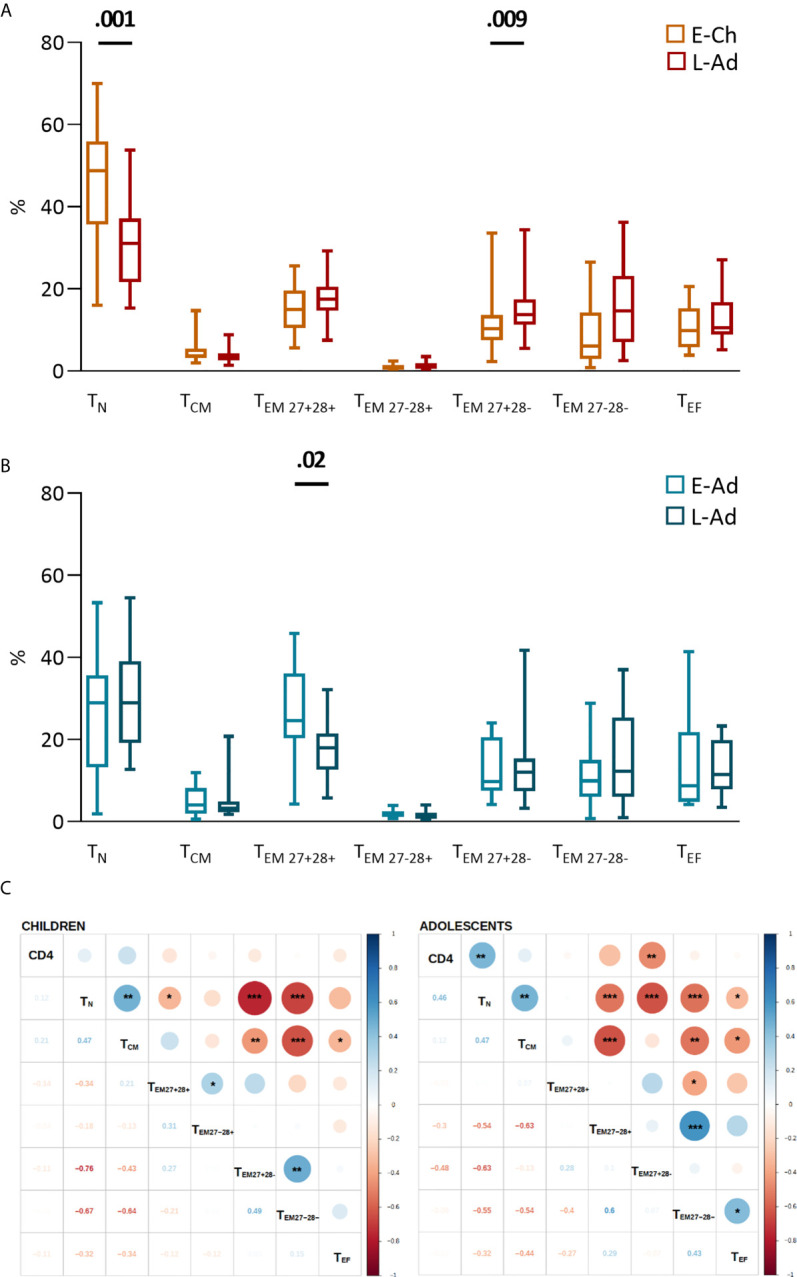
CD8 T-cell differentiation subsets in patients with early and late treatment initiation. T-cell phenotypes were assessed by flow cytometry on fresh whole blood (**Supplementary Material**). The percentage of each subset among total CD8 T lymphocytes is presented for children **(A)** and adolescents **(B)** on box and whiskers plots showing the minimum and maximum values. Early and late treatment groups were compared in Mann-Whitney tests; when significant, *P* values are shown on the graph. Subsets are presented from the least to the most differentiated, and were defined as naive T_N_ (CD45RA^+^CCR7^+^); central memory T_CM_ (CD45RA^-^CCR7^+^); effector memory T_EM27+28+_ (CD45RA^-^CCR7^-^CD27^+^CD28^+^); T_EM27-28+_ (CD45RA^-^CCR7^-^ CD27^-^CD28^+^); T_EM27+28-_ (CD45RA^-^CCR7^-^ CD27^+^CD28^-^); T_EM27-28-_ (CD45RA^-^CCR7^-^ CD27^-^CD28^-^) and effector T_EF_ (CD45RA^+^CCR7^-^CD27^-^CD28-) cells. **(C)** The correlograms present Spearman’s rank correlation coefficients as symbols (upper quadrants) and values. Significant associations are indicated by symbols (*p < .05; **p < .01; ***p < .001). CD4 T-cell counts were used to calculate correlations.

E-Ch had significantly higher CD8T_N_ percentages than L-Ch (48.7% *vs.* 31.0%, *P* = .001), and lower percentages of the most differentiated effector memory cells (CD27^-^CD28^-^CD8T_EM_, 6.1 *vs.* 14.6%, *P* = .009) (**Figure 3A**). E-Ado had significantly higher percentages of the least differentiated CD27^+^CD28^+^CD8T_EM_ than L-ado (24.6% *vs.* 17.9%, *P* = .02), and similar levels of CD8T_N_ (29.0% *vs.* 29.0%, *P* = .50) (**Figure 3B**). CD4 T-cell counts were correlated with CD8 T-cell subset levels (**Figure 3C**). Overall, early ART was associated with the least differentiated CD8 T lymphocytes.

**Figure 4:**
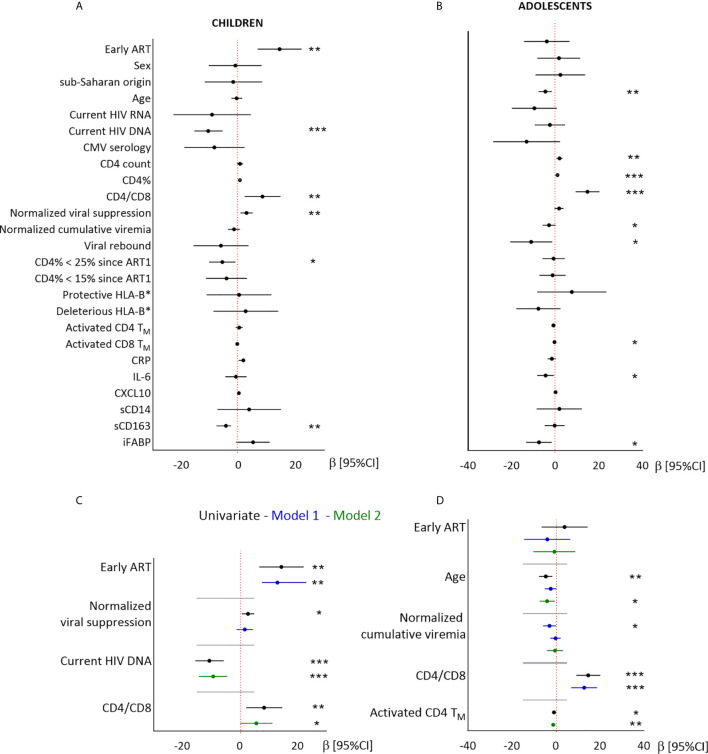
Linear regression analysis of the associations between CD8T_N_ and demographic, virological and immunological factors in children and adolescents. Results from univariate **(A, B)** and multivariate **(C, D)** linear regressions are presented as estimates (β) and 95% confidence intervals. Significant associations are indicated by symbols (*p < .05; **p < .001; ***p < .0001). Multivariate analysis included the covariables indicated on the plot. Estimates are given per year, per 0.1 units of normalized viral suppression, per unit of normalized cumulative viremia, per point of CD4/CD8 ratio, and per 1% of activated CD4T_M_.

In children, higher CD8T_N_ percentages were associated with early ART, a longer normalized duration of viral suppression, lower blood total HIV DNA levels, higher CD4/CD8 ratio, and lower sCD163 levels, but not with either sex or geographic origin (**Figure 4** and **Supplementary Table 3**). Higher CD8T_N_ percentages were independently associated with early ART after adjustment for the duration of viral suppression (model 1), and with higher CD4/CD8 ratio and lower HIV DNA levels (model 2). The associations with CMV serostatus and sCD163 were not significant in multivariate models (data not shown).

In adolescents, higher CD8T_N_ percentages were not associated with early ART, sex or geographic origin, but were significantly associated with younger age, higher CD4/CD8 ratio, lower normalized cumulative viremia, and lower levels of activated CD4 and CD8T_M_ cells, IL-6 and iFABP (**Figure 4** and **Supplementary Table 3**). In multivariate models, higher CD8T_N_ percentages were associated with younger age and higher CD4/CD8 ratio (model 1), or with younger age and lower activated CD4T_M_ cell levels (model 2).

In conclusion, early ART was significantly associated with higher CD8T_N_ percentages in children, but not in adolescents. In both groups, higher CD8T_N_ percentages were associated with higher CD4/CD8 ratio and stronger immune activation.

### Low CD4T_N_ Levels in Adolescents Treated Early may Reflect Limited HIV-Driven T_N_ Production by the Thymus

The lower CD4T_N_ level in E-Ado than in other groups was unexpected. One patient had <5% CD4T_N_ and this very low value was confirmed on the second blood sample. Relative to the reference values for Spain, a second patient had a CD4T_N_ percentage below the 10^th^ percentile, two had values above the 90^th^ percentile, and five had CD4T_N_ percentages between the 10^th^ and 90^th^ percentiles (20).

The E-Ado were born between 1999 and 2003, before early ART was recommended for all newborns. We hypothesized that they might have received early ART because of early diagnosis, reflecting *in utero* infection, or clinical symptoms observed from birth or developing during the first six months of life. We compared E-Ado to E-Ch, who were born between 2004 and 2012, when early ART was the standard of care (**Table 2**). HIV DNA and/or RNA tests were performed during the first week of life for 29 of the participants receiving early treatment: five of six E-Ado and 13 of 23 E-Ch tested positive, indicating prenatal HIV contamination (Fisher’s exact test, *P* = .36). Two patients in each group were diagnosed on the basis of suggestive symptoms (*P* = .25). Two of nine E-Ado and five of 27 E-Ch had at least one CDC stage C event (*P* = .99); these events occurred before the age of six months in both groups. Graphical analyses of CD4T_N_ values as a function of early clinical events and early HIV diagnosis revealed no obvious relationship (**Supplementary Figure 1**). HIV RNA and CD4 levels before, at, and after ART1 initiation were not significantly different between E-Ch and E-Ado (**Table 2**). In conclusion, we found no statistically significant prescription bias (earlier ART related to early and/or severe infection) in E-Ado relative to E-Ch.

**Table 2:** HIV history variables in E-Ch and E-Ado.

Variable	E-Ch	E-Ado	*P* value^a^
	%(n) Median [IQR]	%(n) Median [IQR]	
Positive HIV RNA and/or HIV DNA assay before 7 days of age No Yes	45.0 (10) 55.0 (13)	0.0 (0 100.0 (5)	.36
Diagnosis based on suggestive symptoms No Yes	92.6 (22) 7.4 (2)	78.8(7) 22.2 (2)	.26
Occurrence of a CDC stage C event over lifetime No Yes	81.5 (22) 18.5 (5)	77.8 (7) 22.2 (2)	1.00
Age at the time of the CDC stage C event (months)	2 [1;3]	4 [3;4]	.29
Age at first HIV RNA < 50 copies/mL (days)	302 [150;536]	226 [173;682]	.72
Normalized lifetime spent with HIV RNA ≥ 400 copies/mL	0.16 [0.08;0.24]	0.09 [0.07;0.12]	.11
Zenith HIV RNA before ART1 initiation, log_10_ copies/mL	5.6 [4.1;6.5]	5.5 [5.1;6.0]	.82
HIV RNA at ART1 initiation, log_10_ copies/mL	4.8 [3.4;5.9]	5.5 [4.3;6.0]	.46
Zenith HIV RNA since ART1 initiation, log_10_ copies/mL	3.9 [2.9;5.7]	3.7 [3.0;4.2]	.59
Viral rebound ≥ 400 copies/ml Never Ever	66.7 (18) 33.3 (9)	66.7 (6) 33.3 (3)	1.00
Nadir CD4 T-cell % before ART1 initiation	52 [29;60]	50 [36;56]	.81
CD4 T-cell % at ART1 initiation	52 [29;60]	50 [36;62]	.99
Nadir CD4 T-cell % since ART1 initiation	27 [20;34]	22 [14;26]	.06

a For categorical variables, frequencies, counts and Fisher’s exact test P values are indicated; for quantitative variables, median, interquartile range and Mann-Whitney test P values are indicated.

ART1, first highly active antiretroviral therapy.

HIV replication leads to a depletion of CD4 T cells and drives the *de novo* generation of T_N_ by the thymus in young viremic patients (21–23). We compared E-Ado to L-Ado, whose median percentages of lifetime with HIV RNA levels > 400 copies/mL were 9% and 60%, respectively. The percentage T_RTE_ among CD4T_N_ — an indicator of thymic output —was lower in E-Ado than in L-Ado, but this difference was not significant (**Table 3**). The absolute duration of HIV RNA levels < 400 copies/mL since ART1 was inversely correlated with both CD4T_N_ and CD4T_RTE_ levels in adolescents (Spearman’s rho = -0.402, *P* = .02 and rho=-0.452, *P* = .01). Thus, the low CD4T_N_ levels in E-Ado may reflect limited HIV-driven T_N_ production by the thymus.

**Table 3:** Immune parameters in E-Ado and L-Ado.

Variable	E-Ado	L-Ado	*P* value^a^
	%(*n*) Median [IQR]	%(*n*) Median [IQR]	
% CD4T_RTE_ (among CD4T_N_)	75.0 [74.5;78.8]	78.7 [75.5;83.8]	.27
CMV serology Negative Positive	22.2 (2) 77.8 (7)	4.8 (1) 95.2 (20)	.21
CMV serology, titer	102.0 [73.3;109.0]	104.0 [74.6;124.0]	.28
CMV IFN-γ EliSpot Negative Positive	0.0 (0) 100.0 (5)	12.5 (1) 87.5 (7)	1.00
CMV IFN-γ EliSpot (cells/10^6^ PBMCs)	1 752 [592;3 754]	1 933 [724;3 344]	.94
HLA-DR^+^CD38^+^ CD4T_M_ (% among CD4T_M_)	9.2 [8.3;10.0]	9.9 [9.0;13.3]	.29
HLA-DR^+^CD38^+^ CD8T_M_ (% among CD8T_M_)	11.2 [5.9;14.1]	12.4 [10.2;19.2]	.42
Soluble CD14 (sCD14), µg/mL	1.52 [1.35;1.96]	1.45 [1.25;1.57]	.35
Soluble CD163 (sCD163), ng/mL	287 [279;311]	281 [217;355]	.90
CXCL10, pg/mL	27.1 [19.4;35.1]	40.5 [14.2;124.4]	.39
Interleukin-6 (IL-6), pg/mL	0.53 [0.40;1.33]	0.51 [0.33;1.04]	.72
C-reactive protein (CRP), µg/mL	1.04 [0.73;3.05]	0.57 [0.42;1.31]	.20
Intestinal fatty acid-binding protein (iFABP), ng/mL	0.55 [0.47;1.05]	0.56 [0.46;0.82]	.87

a For categorical variables, frequencies, counts and Fisher’s exact test P values are indicated; for quantitative variables, median, interquartile range and Mann-Whitney test P values are indicated.

IQR, interquartile range; CD4T_RTE_, CD4 recent thymic emigrant; CD4T_N_ %, naive CD4 T lymphocyte percentage; CMV, cytomegalovirus; IFN-γ, gamma interferon; CD4T_M_, memory CD4 T lymphocyte; CD8T_M_, memory CD8 T lymphocyte.

The immune response to pathogens, inflammation and immune activation drive the recruitment of naive lymphocytes to the memory compartment and reduce thymic function (24). CMV prevalence, and the magnitude of CMV-specific serologic and T-cell responses were similar in L-Ado and E-Ado (**Table 3**), and blood samples from all patients tested negative for CMV DNA. These data argue against the low CD4T_N_ levels being driven by an expansion of CMV-specific lymphocytes. E-Ado and L-Ado had similar levels of cellular and plasma markers of immune activation, inflammation, and gut permeability (**Table 3**). Overall, we did not observe higher levels of immune factors specific to E-Ado that could explain their low CD4T_N_ levels.

In conclusion, we considered three explanations for the low CD4T_N_ levels in E-Ado and analyzed the characteristics of the patients and correlations between variables. We found no indication for either a more severe disease profile or a more activated immune profile in E-Ado than in the other groups. Our data suggest that E-Ado have lower CD4T_N_ levels than L-Ado because thymic activity is enhanced to maintain T-cell homeostasis in L-ado.

## Discussion

The ANRS-EP59-CLEAC study included children and adolescents on ART with a good immunovirological status at the time of evaluation. In children, early ART had a beneficial effect for the maintenance of higher proportions of CD8T_N_ lymphocytes and a marginal impact on CD4T_N_ lymphocyte levels. By contrast, early ART had no beneficial effect in adolescents. CD4T_N_ and CD8T_N_ levels were negatively related to cellular activation or gut permeability.

The reported percentages of CD4T_N_ were in the range of those published for age-matched healthy subjects, and only two E-Ado had values below the 10^th^ percentile (20). CD4T_N_ percentages were strongly associated with CD4 T-cell counts, as previously reported (21, 25–27). In our study population, CD4 T-cell counts were in the same range in the early and late treatment groups for both age strata (**Table 1**), reflecting a lessening of the benefits of early ART after several years of treatment (28–31). Most CD4T_N_ are CD4T_RTE_, consistent with the thymus being active in pediatric patients (22, 23, 25, 32).

By contrast to these findings for CD4 T lymphocytes, early ART had a beneficial effect on CD8 T lymphocytes during childhood. E-Ch had median CD8T_N_ values in the same range as uninfected controls, whereas the median values for L-Ch, E-Ado and L-Ado were lower than those of the reference groups (20); eight children and seven adolescents had values below the 10^th^ percentile. E-Ch had a higher CD8T_N_ and CD4/CD8 ratio, and lower total and CD8 T-cell counts than L-Ch (**Table 1**). The dichotomy between robust thymus-driven CD4T_N_ recovery and the persistence of HIV-driven CD8T_N_ loss is a key feature of pediatric HIV infection, reported in ART-treated young adults infected during the perinatal period, until their third decade of life (33). We show here that early ART keeps CD8T_N_ levels high in children, with a possible impact on the future health of the child, because T_N_ lymphocytes have a broad TCR repertoire and a high capacity to respond to new antigens (33).

T_N_ percentages displayed a high level of interindividual variability in all groups. Among the expected factors (17, 34, 35), we found a trend towards lower CD8T_N_ percentages in CMV-infected patients. CD4T_N_ percentages were lower in patients born to mothers of Sub-Saharan origin than in those born to mothers of other geographic origins, although this difference was not significant. We found no association between T_N_ levels and sex.

We document associations between higher immune activation and gut permeability biomarkers and lower T_N_ percentages in at least one of the two age strata. In children, activated CD4T_M_ were more strongly associated with CD4T_N_ than CD4 T-cell counts. iFABP is a marker of intestinal permeability, and impaired mucosal immunity and decreases in thymic output influence each other (3, 36, 37). Higher CD4T_N_ and CD8T_N_ levels were found to be associated with lower iFABP levels, but only in adolescents. No difference in iFABP levels was observed between HIV-1-infected and uninfected infants (38–40), by contrast to what has been observed in adults (41). Thus, the data reported here and in other pediatric studies indicate that gut permeability and its impact on the immune system vary with age.

We found no association between CD4T_N_ and virological history in either children or adolescents. Indeed, E-Ado had the lowest CD4T_N_, despite having the lowest normalized exposure to HIV RNA > 400 copies/ml (median 9% of their lifetime) and low HIV-DNA levels (16). In HIV-infected adolescents who had no access to early ART, CD4T_N_ and T_RTE_ levels were, paradoxically, higher in those with uncontrolled viremia, and HIV replication was correlated with CD4T_N_ levels (21). Our observations are consistent with lower *de novo* thymic T_N_ production in E-Ado than in L-Ado, whose CD4 T-cell compartment was less depleted by HIV replication. Reduced thymic activity may also account for the lack of association between early ART and higher CD8T_N_ in adolescents. Such an association was expected because HIV replication is the main driver of CD8T_N_ loss due to recruitment to the memory pool. However, this association was observed in children but not in adolescents. Thus, at adolescence, thymus-driven T-cell reconstitution may have a stronger influence on naive T lymphocytes than HIV replication.

We identified a small number of adolescents with low T_N_ levels despite long-term viral suppression. Thymic failure may occur in pediatric patients, albeit less frequently than in adults (32). Rare cases of perinatally infected adults on suppressive ART with low CD4 T-cell counts were reported in an Italian study (33). By contrast, low CD4T_N_ counts were mostly associated with poor adherence and active viral replication in an older population of perinatally infected patients living in France (42).

One limitation of this study is a probable survivor bias, particularly for adolescents, potentially resulting in an underestimation of the deleterious impact of late ART initiation. It was not possible to attribute differences (or the lack of difference) between E and L groups definitively to the timing of first ART, because data from an observational study necessarily reflect differences in care according to chronological time and place of birth. Nevertheless, pathophysiological description according to the timing of ART initiation is worthwhile, because it reflects the diversity of patients managed in France and other high-income countries.

In conclusion, we report that, in children over five years of age and adolescents, early ART clearly has a beneficial effect on CD8T_N_ cells. Children in whom treatment was initiated late had high CD4T_N_ levels, probably because of their robust thymic activity. They achieved CD4 T-cell counts similar to those of their peers receiving early treatment, whilst on suppressive therapy. Thus, CD8T_N_ levels, and their principal clinical correlate, CD4/CD8 ratio, are valuable indicators for use in long-term immune reconstitution studies in pediatric patients.

## Data Availability Statement

The raw data supporting the conclusions of this article will be made available by the authors, without undue reservation.

## Ethics Statement

The studies involving human participants were reviewed and approved by Comité de protection des personnes Île-de-France V. Written informed consent to participate in this study was provided by the participants’ legal guardian/next of kin.

## Author Contributions

PF, JLC, CD, VA-F, JW and FB conceived the study and analyzed the data. PF supervised the clinical study. VA-F supervised the virological study. JW supervised the study methodology and statistical analyses. FB supervised the immunological study. TM, DB, and IF performed the immunological tests and analyzed immunological data. JLC performed the statistical analyses. PF, CD, AF, SB, AC, CF, IH, ML, NM, VM, M-DT and FV followed the patients and contributed to clinical data. AH participated to data collection, TW managed the database. FB wrote the manuscript. All authors contributed to the article and approved the submitted version.

## Funding

ANRS (France Recherche Nord et Sud SIDA-HIV Hépatites) funded this study.

## Conflict of Interest

The authors declare that the research was conducted in the absence of any commercial or financial relationships that could be construed as a potential conflict of interest.

## The ANRS CLEAC Study Group

Hôpital Armand Trousseau, Paris: Mary-France Courcoux, Catherine Dollfus, Marie-Dominique Tabone, Geneviève Vaudre; Hôpital Bicêtre, Le Kremlin-Bicêtre: Corinne Fourcade, Josiane Warsazawski, Jérôme Lechenadec, Olivia Dialla, Laura Nailler, Lamya Ait-Si-Selmi, Isabelle Leymarie, Thierry Wack, Alexandre Hoctin, Razika Feraon-Nanache; Centre hospitalier intercommunal de Créteil: Isabelle Hau; Hôpital Delafontaine, Saint-Denis: Cécile Gakobwa; Hôpital Necker-Enfants Malades, Paris: Véronique Avettand-Fenoël, Stéphane Blanche, Marine Fillion, Pierre Frange, Nizar Mahlaoui, Adeline Mélard, Florence Veber, Marie-Christine Mourey; Maternité Port-Royal, Paris: Valérie Marcou; Hôpital Robert Debré, Paris: Albert Faye, Martine Lévine, Sandrine Richard; Pitié-Salpêtrière, Paris: Brigitte Autran, Assia Samri, Mariama Diallo; Hôpital Saint-Louis: Sophie Caillat-Zucman, Kahina Amokrane, Rayna Ivanova-Derin, Centre hospitalier intercommunal de Villeneuve-Saint-Georges: Anne Chacé; Institut Pasteur Paris: Florence Buseyne, Thomas Montange, Damien Batalie, Ingrid Fert, Asier Saez-Cirion, Valérie Monceaux, Daniel Scott-Algara; ANRS: Lucie Marchand, Delphine Lebrasseur, Axel Levier. ANRS (*France Recherche Nord et Sud SIDA-HIV Hépatites*) was the sponsor of the study.
