# Supplementary material for: Impact of Early Versus Late Antiretroviral Treatment Initiation on Naive T Lymphocytes in HIV-1-Infected Children and Adolescents – The-ANRS-EP59-CLEAC Study
Source: Front Immunol. 2021 Apr 22;12:662894. doi: 10.3389/fimmu.2021.662894 (PMC8100053; doi:10.3389/fimmu.2021.662894)
Supplement: Supplementary file 1 [file DataSheet_1.docx]

Supplementary Material

**Supplementary Table 1: ELISA quantification of plasma analytes**

| **Analyte** | **Cat No.** | **Manufacturer** |
| --- | --- | --- |
| C-reactive protein (CRP) | Duoset DY1707 | R&D Systems |
| Interleukin-6 (IL-6) | Quantikine HS600C | R&D Systems |
| CXCL10 | DY06605 | R&D Systems |
| Soluble CD14 (sCD14) | DY383 | R&D Systems |
| Soluble CD163 (sCD163) | DY1607 | R&D Systems |
| Intestinal fatty acid-binding protein (iFABP) | HK406-02 | Hycult Biotech |

# Supplementary Table 2: Univariate analysis of factors associated with CD4T_N_

| **Naive CD4T_N_** | Children *n*=44 | |  | |  | | Adolescents *n*=30 | |  | |  | |
| --- | --- | --- | --- | --- | --- | --- | --- | --- | --- | --- | --- | --- |
| Variable | | Median [IQR] –Pearson’s r  ^a^ | β [95%IC]^a^ | *P* = ^a^ | | Median [IQR] –Pearson’s r | | β [95%IC] | | *P* = | |  |
| Treatment group (Early/Late) | | 61.2 [49.9;68.9]  53.1 [50.0;58.5] | -3.30 [-9.49;2.88] | .29 | | 36.0 [33.2;48.9]  51.5 [43.1;59.1] | | 12.59 [0.80;24.37] | | .04 | |  |
| Sex (Male/Female) | | 53.4 [50.0;65.6]  57.9 [49.9;63.4] | 1.84 [-4.63;8.32] | .57 | | 50.2 [42.1;59.1]  44.1 [34.9;59.1] | | -5.35 [-17.1;6.40] | | .36 | |  |
| Sub-Saharan Africa origin (No/Yes) | | 62.5 [52.5;71.2]  56.5 [49.9;63.2] | -6.03 [-12.9;0.83] | .08 | | 57.1 [42.1;60.2]  45.0 [36.1;59.1] | | 1.40 [-12.4;15.2] | | .84 | |  |
| Age, years | | -0.233 | -1.00 [-2.30;0.30] | .13 | | -0.497 | | -5.50 [-9.21;-1.78] | | .005 | |  |
| Current HIV RNA (< 50 copies/ml / ≥ 50 copies/ml) | | 56.5 [49.3;65.6]  57.1 [56.8;57.5] | 1.16 [-8.55;10.9] | .81 | | 50.8 [42.1;59.1]  40.2 [32.8;55.7] | | -5.20 [-18.3;7.86] | | .42 | |  |
| Total HIV DNA, log_10_ copies/10^6^ PBMCs | | -0.111 | -1.48 [-5.63;2.66] | .47 | | 0.215 | | 4.68 [-3.55;12.91] | | .29 | |  |
| CMV serology (Negative/Positive)^b^ | | 62.3 [50.0;69.1]  56.8 [49.3;63.3] | -3.37 [-10.9;4.20] | .37 | | 36.1 [33.2;74.8]  48.9 [42.1;59.1] | | 0.07 [-19.4;19.55] | | .99 | |  |
| CD4 count, per 100 cells/µL | | 0.317 | 0.76 [0.05;1;48] | .04 | | 0.645 | | 3.07 [1.66;4.48] | | .0001 | |  |
| CD4 percentage | | 0.307 | 0.39 [0.01;0.76] | .04 | | 0.541 | | 0.87 [0.35;1.39] | | .002 | |  |
| CD4/CD8 ratio | | 0.278 | 4.25 [-0.33;8.83] | .07 | | 0.494 | | 12.2 [3.87;20.4] | | .006 | |  |
| Normalized duration of HIV RNA < 400 copies/ml, per 0.1 units ^c^ | | 0.006 | 0.31 [-16.24;16.85] | .97 | | -0.016 | | 0.10 [-2.56;2.75] | | .94 | |  |
| Normalized cumulative viremia since ART1, per 10 units  ^c^ | | -0.017 | -0.08 [-1.60;1.43] | .91 | | -0.125 | | -1.20 [-4.96;2.53] | | .51 | |  |
| Viral rebound ≥ 400 copies/ml (Never/Ever) | | 58.2 [50.0;66.2]  54.7 [49.3;61.6] | -1.88 [-8.78;5.02] | .59 | | 51.5 [42.1;59.1]  42.4 [36.1;48.9] | | -8.86 [-21.1;3.43] | | .15 | |  |
| CD4% < 25% since ART1 (Never/Once/≥2) | | 59.3 [50.0;68.9]  55.5 [50.7;64.5]  52.6 [49.2;59.1] | -0.97 [-9.36;7.43]  -4.21 [-11.16;2.74] | .82  .23 | | 51.5 [42.4;60.0]  43.1 [36.1;74.8]  43.8 [36.1;58.2] | | -0.08 [-20.42;20.25]  -7.09 [-19.31;5.14] | | .99  .25 | |  |
| CD4% < 15% since ART1 (Never/Once/≥2) | | 57.7 [49.9;65.6]  53.4 [50.0;56.8]  51.5 [46.8;65.9] | -4.53 [-19.47;10.42]  -1.60 [-12.43;9.23] | .54  .77 | | 50.0 [42.1;60.0]  43.3 [26.7;48.9]  49.6 [31.8;59.1] | | -11.21 [-30.54;8.12]  -8.11 [-22.61;6.39] | | .24  .26 | |  |
| HLA-DR+CD38+ CD4T_M_ | | -0.375 | -1.05 [-1.86;-0.24] | .01 | | -0.217 | | -0.65 [-1.78;0.48] | | .25 | |  |
| HLA-DR+CD38+ CD8T_M_ | | -0.005 | -0.01 [-0.35;0.33] | .97 | | -0.020 | | -0.02 [-0.51;0.46] | | .92 | |  |
| C-reactive protein (CRP), µg/mL | | 0.213 | 0.84 [-0.36;2.04] | .17 | | -0.369 | | -2.39 [-4.72;-0.06] | | .04 | |  |
| Interleukin-6 (IL-6), pg/mL | | 0.104 | 0.87 [-1.71;3.45] | .50 | | -0.270 | | -3.54 [-8.43;1.36] | | .15 | |  |
| CXCL10, per 100 pg/mL | | 0.011 | 0.01 [-0.12;0.13] | .94 | | 0.014 | | 0.02 [-0.66;0.71] | | .94 | |  |
| Soluble CD14 (sCD14), µg/mL | | 0.097 | 2.44 [-5.40;10.3] | .53 | | 0.110 | | 3.55 [-8.91;16.00] | | .56 | |  |
| Soluble CD163 (sCD163), per 100 ng/mL | | 0.074 | 0.32 [-1.04;1.69] | .63 | | 0.052 | | 0.74 [-4.72;6.19] | | .78 | |  |
| Intestinal fatty acid-binding protein (iFABP), ng/mL | | -0.015 | -0.21 [-4.51;4.09] | .92 | | -0.530 | | -10.92 [-17.65;-4.16] | | .003 | |  |

^a^ Median [interquartile range] or Pearson’s correlation coefficients are shown. Linear regression was used to assess associations between CD4T_N_ and demographic, current status, virological history, genetic and immunologic factors. Estimates, 95% confidence intervals and *P* values are presented;

^b^ Only three adolescents were seronegative for CMV;

^c^ The duration for which HIV RNA < 400 copies/mL and cumulative viremia were calculated since the initiation of ART1 and divided by the time since ART1 initiation

Abbreviations: ART1, first highly active ART; CMV, cytomegalovirus, CD4T_M_, memory CD4 T lymphocyte, CD8T_M_, memory CD8 T lymphocyte.

# Table 3: Univariate analysis of factors associated with CD8T_N_

| **Naive CD8T_N_** | Children *n*=44 | |  | |  | | Adolescents *n*=30 | |  | |  | |  |
| --- | --- | --- | --- | --- | --- | --- | --- | --- | --- | --- | --- | --- | --- |
| Variable | | Median [IQR] –Pearson’s r | | β [95%IC]^a^ | | *P* = | | Median [IQR] –Pearson’s r | | β [95%IC] | | *P* = | |
| Treatment group (Early/Late) | | 48.7 [35.9;55.8]  31.0 [21.9;36.7] | | -14.37 [-22.08;-6.67] | | .001 | | 29.0 [15.9;32.0]  29.0 [20.2;37.6] | | 3.93 [-6.52;14.39] | | .45 | |
| Sex (Male/Female) | | 43.9 [25.8;51.6]  36.0 [29.4;51.9] | | -1.13 [-10.37;8.11] | | .81 | | 26.3 [18.2;37.6]  29.8 [19.1;37.6] | | 1.57 [-8.29;11.43] | | .75 | |
| Sub-Saharan Africa origin (No/Yes) | | 35.1 [27.4;59.6]  38.4 [32.0;49.7] | | -1.86 [-11.96;8.25] | | .71 | | 31.7 [16.8;36.0]  29.0 [18.2;39.2] | | 2.27 [-9.13;13.68] | | .69 | |
| Age, years | | -0.104 | | -0.64 [-2.53;1.26] | | .50 | | -0.506 | | -4.63 [-7.69;-1.57] | | .004 | |
| Current HIV RNA (< 50 copies/ml / ≥ 50 copies/ml) | | 38.4 [32.0;51.9]  25.8 [24.1;35.6] | | -9.25 [-22.75;4.26] | | .17 | | 31.1 [22.6;40.5]  19.5 [13.8;29.1] | | -9.60 [-19.90;0.69] | | .07 | |
| Total HIV DNA, log_10_ copies/10^6^ PBMCs | | -0.555 | | -10.55 [-15.48;-5.62] | | .0001 | | -0.138 | | -2.49 [-9.41;4.42] | | .47 | |
| CMV serology (Negative/Positive)^b^ | | 49.7 [36.5;56.1]  35.6 [27.4;49.7] | | -8.49 [-19.02;2.05] | | .11 | | 39.2 [29.1;53.3]  26.5 [17.5;36.0] | | -13.21 [-28.51;2.09] | | .09 | |
| CD4 count, per 100 cells/µL | | 0.147 | | 0.50 [-0.55;1.55] | | .34 | | 0.471 | | 1.86 [0.51;3.21] | | .009 | |
| CD4 percentage | | 0.249 | | 0.45 [-0.09;0.99] | | .10 | | 0.695 | | 0.93 [0.56;1.30] | | <.0001 | |
| CD4/CD8 ratio | | 0.388 | | 8.42 [2.18;14.7] | | .009 | | 0.723 | | 14.73 [9.29;20.18] | | <.0001 | |
| Normalized duration of HIV RNA < 400 copies/ml, per 0.1 unit ^c^ | | 0.374 | | 2.82 [0.64;5.00] | | .01 | | 0.293 | | 1.66 [-0.43;3.73] | | .12 | |
| Normalized cumulative viremia since ART1, per 10 units  ^c^ | | -0.225 | | -1.56 [-3.65;0.54] | | .14 | | -0.360 | | -2.90 [-5.82;0.01] | | .05 | |
| Viral rebound ≥ 400 copies/ml (Never/Ever) | | 38.5 [32.3;52.2]  33.9 [25.0;42.7] | | -6.15 [-15.80;3.50] | | .21 | | 31.7 [20.7;40.5]  22.6 [12.7;29.0] | | -11.05 [-20.70;-1.39] | | .03 | |
| CD4% < 25% since ART1 (Never/Once/≥2) | | 49.5 [32.6;55.8]  37.0 [31.3;45.6]  31.3 [23.6;43.9] | | -6.92 [-18.25;4.40]  -11.35 [-20.73;-1.97] | | .22  .02 | | 29.0 [20.7;35.5]  29.1 [16.8;53.3]  27.8 [14.9;37.6] | | 4.13 [-13.04;21.30]  -1.52 [-11.84;8.81] | | .63  .77 | |
| CD4% < 15% since ART1 (Never/Once/≥2) | | 37.6 [32.0;52.1]  37.0 [25.8;48.1]  30.3 [23.0;41.2] | | -3.71 [-24.72;17.30]  -8.56 [-23.79;6.66] | | .72  .26 | | 29.0 [20.2;36.0]  29.0 [10.6;54.5]  29.1 [14.9;37.6] | | 2.32 [-14.24;18.88]  -3.16 [-15.58;9.26] | | .78  .61 | |
| HLA-DR+CD38+ CD4T_M_ | | 0.053 | | 0.21 [-1.03;1.45] | | .73 | | -0.346 | | -0.86 [-1.76;0.04] | | .06 | |
| HLA-DR+CD38+ CD8T_M_ | | -0.286 | | -0.44 [-0.91;0.02] | | .06 | | -0.356 | | -0.37 [-0.74;0.01] | | .05 | |
| C-reactive protein (CRP), µg/mL | | 0.010 | | 0.06 [-1.69;1.80] | | .95 | | -0.306 | | -1.64 [-3.61;0.33] | | .10 | |
| Interleukin-6 (IL-6), pg/mL | | -0.074 | | -0.88 [-4.56;2.80] | | .63 | | -0.413 | | -4.49 [-8.32;-0.66] | | .02 | |
| CXCL10, per 100 pg/mL | | 0.233 | | 0.13 [-0.04;0.31] | | .13 | | 0.039 | | 0.06 [-0.51;0.62] | | .84 | |
| Soluble CD14 (sCD14), µg/mL | | 0.104 | | 3.72 [-7.41;14.9] | | .50 | | 0.066 | | 1.77 [-8.58;12.1] | | .73 | |
| Soluble CD163 (sCD163), per 100 ng/mL | | -0.408 | | -2.56 [-4.37;-7.77] | | .006 | | -0.030 | | -0.35 [-4.87;4.18] | | .88 | |
| Intestinal fatty acid-binding protein (iFABP), ng/mL | | 0.258 | | 5.07 [-0.83;10.9] | | .09 | | -0.439 | | -7.49 [-13.4;-1.56] | | .02 | |

^a^ Median [interquartile range] or Pearson’s correlation coefficients are shown. Linear regression was used to assess associations between CD4T_N_ and demographic, current status, virological history, genetic and immunologic factors. Estimates, 95% confidence intervals and *P* values are presented;

^b^ Only three adolescents were seronegative for CMV;

^c^ The duration for which HIV RNA < 400 copies/mL and cumulative viremia were calculated since the initiation of ART1 and divided by the time since ART1 initiation

Abbreviation: ART1, first highly active ART; CMV, cytomegalovirus, CD4T_M_, memory CD4 T lymphocyte, CD8T_M_, memory CD8 T lymphocyte.

**Supplementary figure 1: CD4T_N_ levels according to signs of early HIV disease or infection.** T-cell phenotypes were assessed by flow cytometry on fresh whole blood. The percentage of T_N_ (CD45RA^+^CCR7^+^ among total CD4 T lymphocytes) is shown for E-Ch and E-Ado. Patients with early clinical disease (CDC stage C before the age of one year) are highlighted with gray symbols and those with an HIV DNA or RNA assay performed in the first week of life are outlined in color (red for a positive assay; blue for a negative assay).

Study of lymphocyte phenotypes

We stained 100 µl of whole blood with the following mixtures of antibodies, in 5 mL tubes (Becton Dickinson, 352054). Cells were incubated for 30 min at room temperature in the dark. We then added 3 mL of lysis kit (eBioscience, 00-5333-57) and mixed samples by low-speed vortexing. The cells were incubated for 15 min at room temperature, centrifuged for 6 min at 500 x *g*, washed once in 2 mL PBS, resuspended in 0.3 mL PBS and immediately analyzed.

The data presented in the manuscript were obtained under the following three sets of staining conditions.


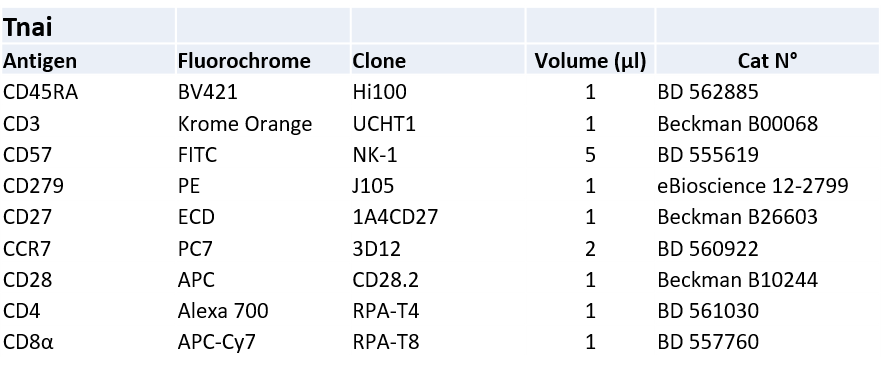


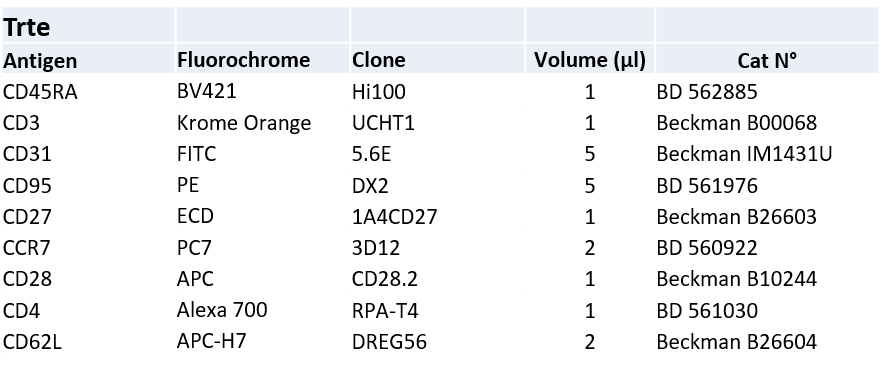


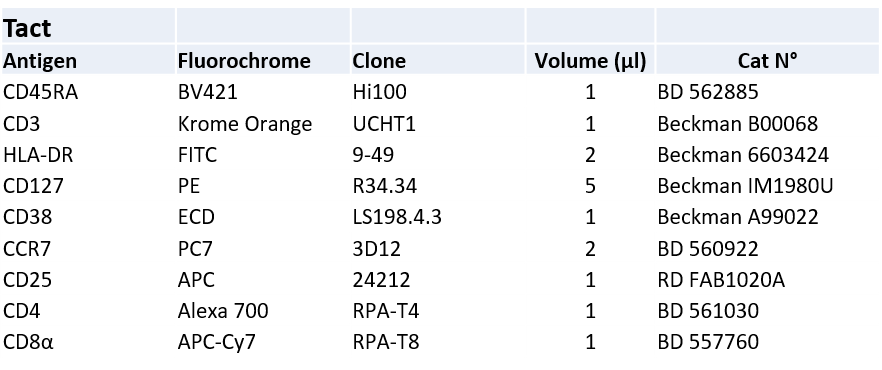


Cells were analyzed with a Gallios flow cytometer (Beckman-Coulter). The detectors used for each antibody specificity are described in the table.

**
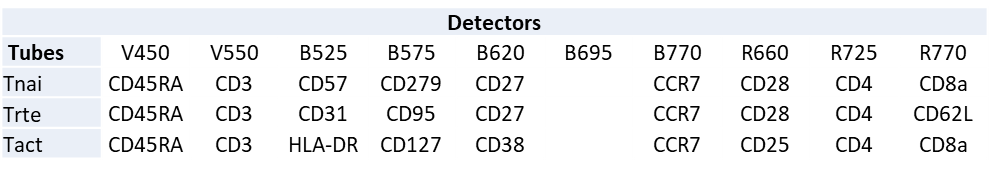
**

**Gating strategy.** Kaluza software was used for analysis.

STEP 1: Lymphocytes were selected by sequential application of the following gates (from left to right):

- A time-FSCINT gate to define events acquired at a constant flow speed;

- An SSCINT-FSCINT gate to define lymphocytes;

- An SSCINT-SSPEAK gate to define singlet events;

- An FSTOF-FSCINT gate to define singlet events, because the SINGLE-1 gate includes some doublets.


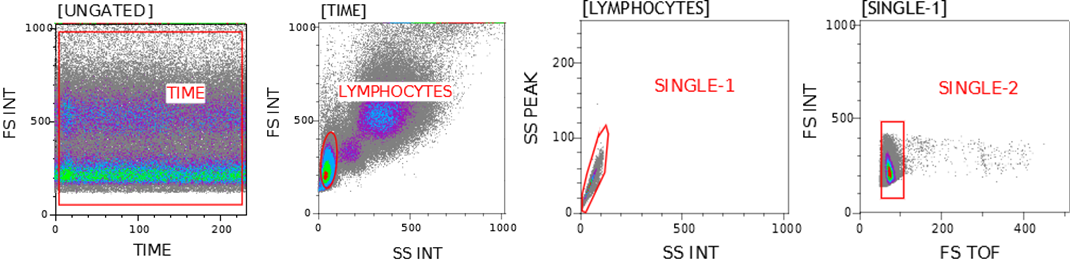


STEP 2: CD4 and CD8 T lymphocytes and their CD45RA/CCR7 subsets were selected by sequential application of the following gates (from left to right):

- A CD3-FSINT gate applied to events selected by SINGLE-2 to define T lymphocytes;
- CD4^+^CD8β^-^ and CD4^-^CD8β^+^ gates applied to CD3 lymphocytes to define CD4 and CD8 T lymphocytes;
- Quadrants to define 4 subsets based on CD45RA and CCR7 expression.

STEP 3: CD4 T lymphocyte subsets labeled in the Tnai tube were further defined by application of:

- A CD27^+^CD28^+^ gate on CD45RA^+^ (CD45RA^+^R7^+^ OR CD45RA^+^R7^-^) cells to define naive T cells (T_N_);
- A CD27^-^CD28^-^ gate on CD45RA^+^ (CD45RA^+^R7^+^ OR CD45RA^+^R7^-^) cells to define effector T cells (T_EF_);
- A CD27^+^CD28^+^ gate on CD45RA^-^R7^-^ cells to define transitional memory T cells (T_TM_);
- A CD27^-^CD28^+^/^-^ gate on CD45RA^-^R7^-^ cells to define transitional memory T cells (T_EM_).


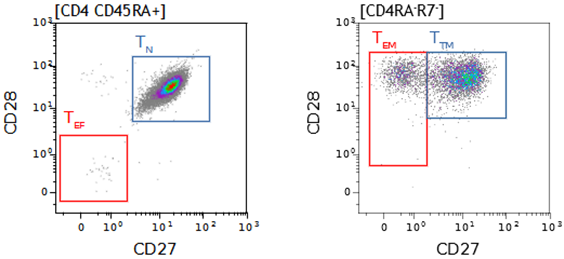


STEP 4: CD8 T lymphocyte subsets labeled in the Tnai tube were further defined by application of:

- A CD27^+^CD28^+^ gate on CD45RA^+^ (CD45RA^+^R7^+^ OR CD45RA^+^R7^-^) cells to define naive T cells (T_N_);
- A CD27^-^CD28^-^ gate on CD45RA^+^ (CD45RA^+^R7^+^ OR CD45RA^+^R7^-^) cells to define effector T cells (T_EF_);
- Quadrants to define four T_EM_ subsets based on CD27 and CD28 expression.

STEP 5: CD4 T lymphocytes subsets labelled in the Trte tube were further defined by application of

- A CD31+CD95- gate on CD45RA+CCR7+ cells to define recent thymic emigrants (T_RTE_);
- A CD31-CD95- gate on CD45RA+CCR7+ cells to define CD31- naive T cells (CD31^neg^T_N_);
- A CD95+ gate on CD45RA+CCR7+ cells to define stem cell memory T lymphocytes (T_SCM_).


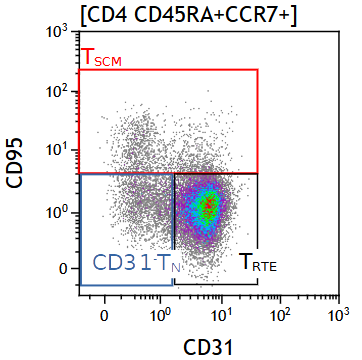


STEP 6: CD4 and CD8 T lymphocytes in the Tact tube were further defined by application of an HLA-DR^+^CD38^+^ gate on the memory (not CD45RA^+^R7^+^) cells to define activated cells.
